# Experiences of telehealth in general practice in Australia: research protocol for a mixed-methods study

**DOI:** 10.3399/BJGPO.2021.0187

**Published:** 2022-01-26

**Authors:** Sarah J White, Amy Nguyen, Peter Roger, Tim Tse, John A Cartmill, Simon Mark Willcock

**Affiliations:** 1 Centre for Social Impact, UNSW, Kensington, Australia; 2 Macquarie Medical School, Faculty of Medicine, Health and Human Sciences, Macquarie University, Sydney, Australia; 3 St Vincent’s Clinical School, UNSW Medicine, UNSW Sydney, Sydney, Australia; 4 Centre for Health Systems and Safety Research, Australian Institute of Health Innovation, Macquarie University, Sydney, Australia; 5 Department of Linguistics, Faculty of Medicine, Health and Human Sciences, Macquarie University, Sydney, Australia; 6 Department of Primary Care, Faculty of Medicine, Health and Human Sciences, Macquarie University, Sydney, Australia; 7 Macquarie Medical School, Faculty of Medicine, Health and Human Sciences, Macquarie University, Sydney, Australia

**Keywords:** Australia, communication, COVID-19, digital health, general practitioners, referral and consultation, telehealth, telemedicine

## Abstract

**Background:**

Owing to the COVID-19 pandemic, the use of telehealth has expanded rapidly. However, little is known about the impact of delivering care through telehealth on communication between clinicians and patients. At an interactional level, the ways in which clinicians establish rapport and connection with their patients in telehealth consultations is not well understood.

**Aim:**

This study will explore interactional practices of GPs and patients in telehealth consultations to develop evidence-based resources to improve communication.

**Design & setting:**

The study will be conducted within the Australian general practice setting.

**Method:**

Conversation analysis and sociolinguistic discourse analysis of recorded telehealth consultations will provide direct evidence of specific elements contributing to successful and less successful instances of telehealth communication. This analysis will be complemented by co-design techniques such as qualitative and reflective interviews, and collaborative workshops with telehealth users including both GPs and patients.

**Conclusion:**

Effective communication is critical for telehealth consultations and is central to achieving optimal clinical outcomes and patient satisfaction. Evidence-based guidelines encompassing effective telehealth communication strategies will be co-developed with end-users in this study.

## How this fits in

Owing to the COVID-19 pandemic, there is a rapidly growing body of work on telehealth. By using a mixed-methods, multi-stage approach, this study will create a comprehensive picture of how telehealth works in practice, what communication strategies are unique to telehealth, and how doctors and patients experience telehealth. This will be translated into evidence-based guidelines and training resources, helping to bridge the gap between evidence and practice.

## Introduction

Telehealth is not new to Australia, having long been used — albeit to a limited extent — in rural and remote settings.^1^ However, it is new to the great majority of Australian doctors, with the Australian government introducing temporary new items on the Medicare Benefit Schedule (Australia’s system for providing funding for medical services) in response to the COVID-19 pandemic. Since this rapid escalation in the availability of telehealth services, ongoing (and constantly changing) isolation and physical distancing restrictions in different parts of the country have made telehealth an imperative.^2^ There have been limited opportunities to support this major transition, particularly regarding approaches to communicating effectively. Clinicians have largely learnt to manage telehealth interactions by trial and error, and by accessing *ad hoc* web resources. The shift in relationship-building and other communication practices from face-to-face consultations to telephone or video calls may significantly impact on patient satisfaction and clinical outcomes.

While there is little information on interactions between GPs and patients during telehealth consultations, existing studies provide evidence of challenges relating to communication, and establishing rapport and trust through a digital connection.^3,4^ These issues are largely around difficulties with clinicians being unable to perceive non-verbal cues.^5^ Interactional research on telehealth has been minimal, although more interest is developing in the space with work on decision making^6^ and examination using video-conferencing.^7^ A systematic review^8^ found only 45 articles exploring communication during telehealth and revealed that training in communication skills specifically for telehealth delivery was consistently mentioned as being required across various disciplines and settings.

Existing advice regarding telehealth tends to focus on the technological and practical sides of the phenomenon with little guidance about the communication itself.^9^ Advice on avoiding distractions and ensuring sufficient signal strength is important, but the real hazards lie at the deeper communicative levels of the interaction. Advice to *‘use strategies and evidence-informed practices to reflect the standard of care expected in a face-to-face consultation, as far as possible’* ^9^ is to be expected from a guideline and, taken at face value, appears to be sensible. Such advice, however, is ultimately naïve. The modalities are very different and so will be the hazards and pitfalls. For example, in pilot research on telehealth in specialist care, the ability to raise new concerns or questions was more limited in telehealth consultations, with more reliance on a patient’s initiative than opportunities provided by the doctor.^10^

This article describes the research protocol for a project focused on telehealth in general practice in Australia. The project has three interrelated aims:

1. To systematically analyse interactional practices of GPs and patients in telehealth consultations to develop evidence-based resources based on interactional elements and patterns associated with desired consultation outcomes.
2. To examine how telehealth influences both GPs’ and patients’ perceptions of the clinical relationship to ensure that guidelines developed help both parties to build positive clinical relationships through this novel mode of communication.
3. To determine the key concerns of GPs in relation to communication and telehealth to inform new evidence-based advice and resources that directly address these concerns.

The project's interdisciplinary approach, which involves close collaboration between medical practitioners, clinical communication, and health services researchers, will ensure that the research is driven from the outset by the needs of practitioners, and that it generates tangible outcomes that are relevant in guiding improvements to telehealth practice.

This research will drive a fundamental shift in the way that best practice standards in telehealth are determined. Importantly, it will bridge the gap between ‘work-as-imagined‘ and ‘work-as-done’^11^ in the implementation of telehealth in Australian general practice. Although telehealth guidelines for medical practitioners exist, such guidelines have often been compiled quickly in response to the rapid roll-out of telehealth and therefore are based on the kinds of problems that could be ‘imagined’, rather than being grounded in evidence that comes from the systematic study of actual experience.

Effective communication is essential to safe clinical practice, and the outcomes of the proposed research will ensure that there is relevant, evidence-based advice available for best practice in the ongoing use of telehealth. The recent research literature on telehealth often alludes to the challenges in building rapport and trust. Grenda *et al* ^12^ note that the *‘patient–physician relationship has traditionally been developed during face-to-face interactions, which will be a shift from our current culture* *‘*. What is missing from the current evidence base, however, is information on precisely *how* these trusting clinical relationships can be best built and maintained through the telehealth medium. This is a key outcome that the current study will provide.

## Method

The study will use a mixed-methods approach, employing a variety of connected data sources and both qualitative and quantitative methods. There are three phases in the project. The project will have two data collection and analysis phases to gain a deep understanding of how communication varies between face-to-face and telehealth consultations, and in what contexts. The third phase will focus on telehealth guideline and training development.

### Phase 1 – Interaction analysis of telehealth consultations

Five GPs will be recruited to the study, and each will be asked to record at least six telehealth consultations to reach a sample size of at least 30 telehealth consultations. In addition, five patients (one per GP recruited) who regularly have telehealth consultations with the same GP will be asked to participate in a tracking component, with every consultation between the GP and that patient during a 3-month period being collected. It is expected that this will yield another 30 consultation recordings. Participants will also be invited to complete post-visit surveys regarding their perceptions of the visit and experience of telehealth.

The consultations will be recorded through video-conferencing software or, for audio-only calls, using mobile phone audio-recording functions or an external digital recorder while the phone is on speakerphone mode. Most data will be recorded through video-conference. This study will deliberately forgo the information that could be gained through additional video coverage of the participants as it is of little relevance to the narrower focus of attention on telehealth, and would be difficult to achieve given current COVID-19 proximity restrictions. Post-visit surveys will be sent via email or text and will have a 72-hour time limit for completion.

Methods of interaction analysis will be used, specifically conversation analysis^13^ and sociolinguistic discourse analysis.^14^ Analysing recorded consultations preserves the features of the talk used, which means it is not necessary to rely on memory and reporting, and possible to repeatedly observe the talk as it actually occurs. Recordings also allow for collaborative analysis. Validity in these methods is established through multiple processes, such as interactional evidence for effectiveness of communication practices (for example, how did the communication phenomenon being described impact conversational flow), team analytic process with individual and group analyses and consensus, and relational analysis with other data types (for example, surveys and interviews). These methods are well established in medical consultation research, with a strongly established theoretical and applied research foundation.^15,16^

The post-visit survey includes the use of two validated surveys, the CARE Patient Feedback Measure^17^ and the Telehealth Usability Questionnaire.^18^ These surveys were chosen because they provide insight into patient perceptions of the consultation and of the impact of telehealth on the consultation. Analysis of the survey data will be conducted with reference to the linked consultations and the analyses of those interactions, along with statistical analysis of the aggregated quantitative data and thematic analysis of qualitative data across the surveys.

### Phase 2 – Qualitative interviews with GPs and patients who have used telehealth

Participants from Phase 1 will be invited to participate in Phase 2 of this project. Additional participants will be recruited through specific calls for participation through professional networks, social media, and participating clinics.

In this phase, post-Phase 1 interviews will be conducted with the participating GPs to identify their key areas of strength and concern in relation to telehealth, and to explore findings from Phase 1; for example, situations where key concerns are more likely and how concerns can be addressed. Between 10 and 20 interviews will be conducted with GPs. The five tracking patients from Phase 1 will also be interviewed to explore experiences of telehealth and how they connect with the patient’s perspective of the clinical relationship.

Semi-structured interviews will provide insight into the personal experiences and perspectives of users of telehealth consultations; clinicians and patients as users are experts in their own lived experiences. A thematic analysis approach^19^ will be used to identify themes and subthemes raised by interview participants. Thematic analyses of interviews with end users has been used in qualitative research methodologies, in particular in the design of health technologies.^20^ Question guides will be iteratively reviewed as more interviews are conducted to ensure that questions cover important arising themes from prior interviews. To ensure that all perspectives are covered, interviews will be conducted until thematic saturation (the point at which no new themes arise) is reached. Additional reflexive analysis interviews,^21^ which include viewing vignettes of videos collected in Phase 1, will be conducted with the five participating GPs to explore their perceptions of and reflections on their own communication. Reflexive analysis draws on the expertise of the participants within the consultations and provides insight into the motivations for particular communication choices as they occurred. This complements both the interaction and thematic analysis, connecting practice, attitudes, and reflection. This is an established research method in health care and beyond.^22^

### Phase 3 – Intervention: telehealth guideline and training resource development

Drawing on findings from Phases 1 and 2, easy-to-use advice and training resources will be created for GPs to support them in engaging with telehealth effectively. Using a co-design approach^23^ which is designed to ensure healthcare improvements and their implementation are tailored to meet the unique needs identified by end users, GPs and relevant stakeholders will be involved in the design of guidelines and training resources based on the analyses of both recorded telehealth consultations and interviews. These guidelines and related training resources will be developed in collaborative workshops with the recruited GPs as well as other relevant stakeholders (including telehealth patients and health consumers) to ensure feasibility. These will be evaluated through participant surveys with a follow-up plan for a future intervention study to assess how evidence-based training influences practice.

As the intervention phase includes development of training resources, action research^24^ will be used to support iterative refinement. Validity in action research is established through use of multiple data points and collaborative analytic approaches. As with the methods described above, the rigorous, collaborative, and transparent application of the research methods allow for the establishment of their trustworthiness.

## Discussion

While telehealth has been used over the past decade, it was not until 2020 that it became widely available in Australia, an imperative driven by the COVID-19 global pandemic. Even after the peak of the pandemic passes, telehealth is likely to continue to be an important part of health care into the future, as the benefits of this new normal begin to be discovered. The evidence produced through this project will highlight the strengths, barriers, and concerns of GPs and patients in how they have experienced telehealth. This will contribute much-needed depth to ongoing health policy discussions.

Effective communication is arguably even more crucial for telehealth consultations as all elements are conducted through interaction without aspects of physical examination. Doctors are learning ’on the job‘ how to translate their face-to-face skills to deliver care to patients over the phone, as there is insufficient evidence about how telehealth interactions work in practice. This study will determine important parameters of effective telehealth communication strategies and, based on this evidence, develop robust guidelines and resources for GPs about best practice for telehealth communication. The fact that these guidelines will be developed in close consultation with GPs for use in general practice will ensure their relevance to real-world telehealth practice situations. Importantly, it will ’level the field’ in terms of quality of care, providing resources to ensure that telehealth care in Australian general practice is of a high standard that is comparable to in-person consultations.
